# Ionic liquid-based enzyme-assisted extraction of chlorogenic acid from *Flos Lonicera Japonicae*

**DOI:** 10.1186/s40643-017-0175-3

**Published:** 2017-10-24

**Authors:** Yang Sun, Song Ding, He Huang, Yi Hu

**Affiliations:** School of Pharmaceutical Sciences, State Key Laboratory of Material-Oriented Chemical Engineering of Nanjing Tech University, Nanjing, 211816 China

**Keywords:** Ionic liquid, Enzyme-assisted, Chlorogenic acid, *Flos Lonicera Japonicae*

## Abstract

**Background:**

In recent years, ionic liquids and enzymes have been widely used in the separation and extraction processes of natural products. Chlorogenic acid (CGA) has important biological and pharmacological activities. It is significant to develop a green and efficient method to extract GCA from Flos Lonicera japonica (FLJ) by integrating the advantages of the ionic liquids and enzymes.

**Results:**

The optimal type of enzyme and ionic liquid was screened. Pectinase in [C6mim] Br aqueous phase was demonstrated to be an ideal combination. The parameters including extraction time, extraction temperature, pH, enzyme amount, and IL concentration were optimized systematically. Scanning electronic microscopy of FLJ samples demonstrated that pectinase and ionic liquid disposal both obviously facilitated the extraction process by destroying the structure of cell wall. Circular dichroism spectroscopy showed that ionic liquid enhanced the activity of the pectinase by altering its secondary structure.

**Conclusions:**

Compared with previous reported methods, ionic liquid-based enzyme-assisted extraction of GCA from FLJ was proved to be efficient and practical, offering a higher yield in a shorter time. A novel process was proposed for the extraction of active component from natural resources.
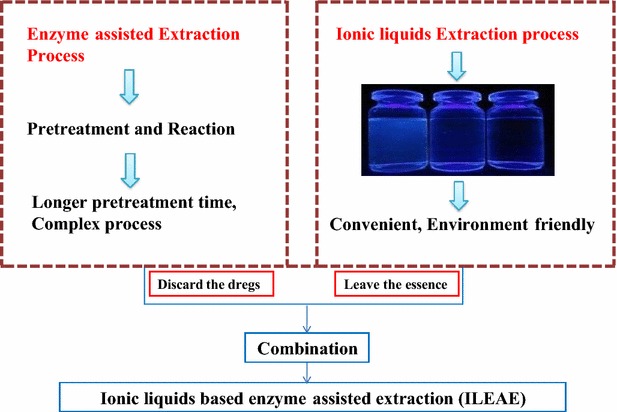

## Background

*Flos Lonicera japonica* (*FLJ*) is widely distributed in China and officially listed in the Chinese pharmacopoeia (Xiang and Ning 2008). It was reported to have various biological activities such as antiviral, antioxidant, and anti-inflammatory and widely used in treating exopathogenic wind-heat, epidemic febrile diseases and some infectious diseases such as SARS coronavirus and swine H1N1 flu virus (Shang et al. 2011; Wang and Weller 2006). Chlorogenic acid (CGA) is the major bioactive components of *FLJ*, and had been reported to have various biological and pharmacological activities such as antioxidant, anti-inflammatory, and anti-carcinogenic activities (Wu et al. 2012; Shin et al. 2015). A series of methods had been reported for the extraction bioactive CGA from plant materials. In conventional methods, CGA extraction was mainly achieved by water extraction (WE) (Zheng et al. 2006; Wu et al. 2006) or refluent ethanol extraction (REE) (Lan and Zhang 2005). Some disadvantage of these methods were fraction of oxidation, hydrolysis, and ionization due to excessive extraction time and relatively higher temperature (Duarte et al. 2010). Nowadays, microwave-assisted extraction (MAE) (Zhang et al. 2008) and ultrasound-assisted extraction (UAE) (Hu et al. 2009) had been developed as the feasible extraction methods. Although these two extraction methods greatly lessen the extraction time, it still can’t meet the satisfaction of green chemistry for the consumption of organic solvents (Zhang et al. 2011).

In this work, we come up with a method to use ionic liquid aqueous phase to enhance the penetrability of the solvent over the extraction process and utilize the characteristics of enzymatic hydrolysis on cell wall to enhance efficiency and extraction yield of CGA from *FLJ*. The optimized ILEAE method was investigated and the extraction mechanism by ILEAE was discussed by scanning electron microscopy and circular dichroism spectroscopy.

## Experimental

### Reagents and materials

*Flos Lonicera japonica* was obtained from Xinyi Honeysuckle Agricultural Development Co., Ltd. (Xin yi, China). *FLJ* was crushed and dried in the hot air oven before extraction. The standard sample of CGA was purchased from Aladdin (Shanghai, China) and its purity was ≥ 98%. All ionic liquids were bought from Shanghai Cheng Jie Chemical Co., Ltd. (Shanghai, China). Cellulase, pectinase, dextranase, and xylanase were purchased from Jiangsu Rui Yang Biotechnology Co., Ltd. (Jiangsu China). Other reagents were analytical grade and used without further treatment. Deionized water was used to prepare the sample solutions.

### Analysis method

The chromatographic system (Dalian, China) consisted of ultimate 3000 autosampler, ultimate 3000 pump, and ultimate 3000 variable wavelength detector. Chromatographic separation was performed on a Amethyst C18-H (4.6 mm × 250 mm, 5 μm) reversed-phase column (Sepax Technologies, Inc.). For HPLC analysis, gradient elution was performed using methanol–water as the mobile phase. CGA was separated by UV detector at a wavelength of 327 nm. Injection volume was 10 ml, flow rate was 1.0 ml/min and column temperature was maintained at 25 °C. The CGA standard curve showed a good linear relationship within the scope of 0.01–1 mg/ml. The standard formula for extraction yield of CGA was $y = 0. 9 9 9x + 1. 2 9 6$ (*R* = 0.999, *x*: concentration, g/ml; *y*: the peak area).

### Optimization of extraction systems

In the preliminary work, the components of extraction solution had been found having significant influence on extraction yield of GCA. Then, several experiments were carried out to investigate on solution components.

### Screening the enzyme type

Calculated amounts of cellulase, pectinase dextranase, and xylanase were weighed 20 mg, respectively and an amount of 0.25 mol/l [C4mim]Br solvent was added to attain concentration of 0.5 mg/ml. The ionic liquid solvent pH 4.0 was prepared for further usage. 2.0 g dried samples were put into 40 ml of the enzymatic ionic liquid solvent and then put in a shaking bath at temperature of 40 °C for 2 h, individually. After the reaction, the extract was filtrated and diluted for subsequent HPLC analysis.

### Screening the ionic liquid type

In the original step, five different anions including Br^−^, Cl^−^, NO3^−^, BF_4_^−^, and PF_6_^−^ and cations including [C2mim]^+^, [C4mim]^+^, [C6mim]^+^, [C8mim]^+^, and [C10mim]^+^ with different length of carbon chains of glyoxaline were examined. Enzyme solution was added into 0.25 M ionic liquid solution to attain a concentration of 0.5 mg/ml, respectively. The ionic liquid solvents were prepared to adjust and attain solutions with pH of 4.0. 2.0 g dried samples were combined with 40 ml of the selected four enzymatic ionic liquid solution, individually and reacted under mixing 150 rpm (on a shaking bath) at temperatures of 40 °C for 2 h. After the reaction, the extract was filtrated and diluted for the further HPLC analysis.

### Determination of pectinase activity

The pectinase activity was determined by hypoiodite sodium method according to Eq. (1). In this formula, *B* was Na_2_S_2_O_3_ consumption of control sample; *A* was Na_2_S_2_O_3_ consumption of control sample; *N* was equivalent concentration of Na_2_S_2_O_3_; 0.51 was a constant meaning that 1 mg equivalent Na_2_S_2_O_3_ equal to 0.51 mg equivalent free galacturonic acid; *S* was the total reaction liquid volume for keeping warm; *E* was used enzyme volume; *t* was the holding time; *M* was absorbing reaction quantity. The amount of enzyme produced by enzymatic reaction of 1 μg equivalent of galacturonic acid per minute was 1 enzyme activity unit (U).

1 $${\text{Pectinase activity}} = \frac{{\left( {B - A} \right) \times N \times 0.51 \times S \, }}{ \, E \times t \times M}$$

### Ionic liquid-based enzyme-assisted extraction (ILEAE)

Pectinase was put into ionic liquids to attain IL-enzyme aqueous solution of concentrations (0.25–2.0 mg/ml). IL solutions were prepared to adjust pH values (3.0–5.0) for attaining the proper pH of extraction solution. 2.0 g dried sample was put into the IL-enzyme aqueous solvent and then it was put on a shaking bath for 0–4 h at 20–70 °C. After ILEAE, the extracts were filtrated and then diluted through a 0.45 μm filter for further HPLC analysis. All experimental steps were performed in duplicate.

### Scanning electron micrographs (SEM)

TM3000 benchtop scanning electron microscopy (Hitachi Co., Ltd. Japan) was applied to examine the effect of IL-enzyme solutions on the structural changes of the plant cells after treatment. The samples were fixed on adhesive tape and examined under high vacuum condition at a voltage of 15.0 kV (10 μm, 1500 magnification).

### Circular dichroism (CD) spectroscopy

Circular dichroism (CD) spectra were recorded on a JASCO-J810 Spectropolarimeter (Jasaco Co., Ltd. Japan) in a cell with 1 cm light path length at 25 °C. The scanning wavelength was 200–270 nm and the scanning rate was set at 50 nm/min. The CD spectra were expressed in terms of the average residue molar ellipticity in units of deg cm^2^ dmol^−1^. After obtaining CD spectra, the secondary structure of lipase was calculated using CDNN (version 2.1) which was distributed by Applied Photophysics.

## Results and discussion

### Sieving of enzyme and ionic liquid type

**Fig. 1:**
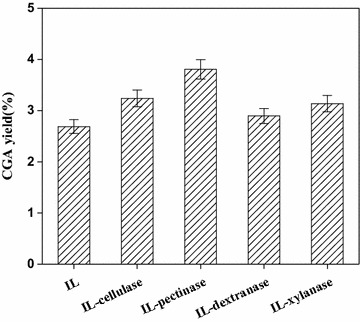
Effect of different enzyme types on the extraction yield of CGA from *FLJ*

We considered the kinds of enzymes of literature commonly used in the enzymatic hydrolysis of the cell wall and finally selected four of the most representative enzymes including pectinase, cellulase, dextranase, and xylanase. The operation conditions were as follows: extraction temperature 40 °C, liquid–solid ratio 20 ml/g, enzyme concentration 0.5 mg/ml, pH 5.0, and extraction time 2 h in the 0.25 M [C4mim]Br aqueous solvent. From Fig. 1, the extraction yields were much higher than that without enzyme treatment, and the extraction yield of treatment with pectinase was higher than those with other enzyme types.

**Fig. 2:**
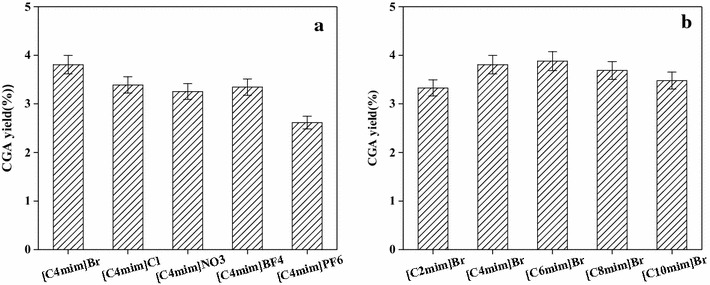
Effect of ionic liquid anions (**a**) and cations (**b**) on the extraction yields of CGA from *FLJ*

As a green solvent, ionic liquids had many potential applications and were applied in chemical industry on extraction of a variety of natural products (Jing et al. 2008; Yang et al. 2016), the component of the ionic liquids had a great effect on its physicochemical properties and may have a strong impact on the target analyte of the extracted yield. In order to sieve the better combination of anions and cations, both of them were investigated, respectively. We investigated several common anions on the basis of literature and finally screened out five anions including Br^−^, Cl^−^, NO_3_^−^, BF_4_^−^, and PF_6_^−^ (Zhang et al. 2011; Ma et al. 2011; Merlet et al. 2017). As can be seen from Fig. 2(a), four kinds of hydrophilic anions showed better extraction effect, while the extraction yield of hydrophobic PF6^−^ was relatively poor. In the four hydrophilic anions, the halogen ions exhibit the best extraction yield especially Br^−^, which may be due to the combination of the halogen ions with the H^+^ in the cellulose to disintegrate the internal structure of cellulose. This result showed that the extraction rate of CGA depends on the type of anion which has been reported (Liu et al. 2016). Based the same cation of Br^−^, different groups of cations including [C2mim]^+^, [C4mim]^+^, [C6mim]^+^, [C8mim]^+^, and [C10mim]^+^ were examined and the results of the evaluation are shown in Fig. 2. From Fig. 2, the extraction yield of CGA from ethyl to hexyl is obviously increased, it may be because CGA can be better dissolved in the lipophilic IL; the extraction yield of CGA decreased from hexyl to decylate may be due to the hydrogen bond and hydrophobic interaction of ionic liquids affecting the dissolution of CGA.

### Optimization of the ILEAE process

In the initial process, we investigated the enzyme types and ionic liquid types. Subsequently, important factors affecting extraction yield including extraction time, extraction temperature, pH, enzyme concentration, and IL concentration were studied.

### Effect of extraction time

**Fig. 3:**
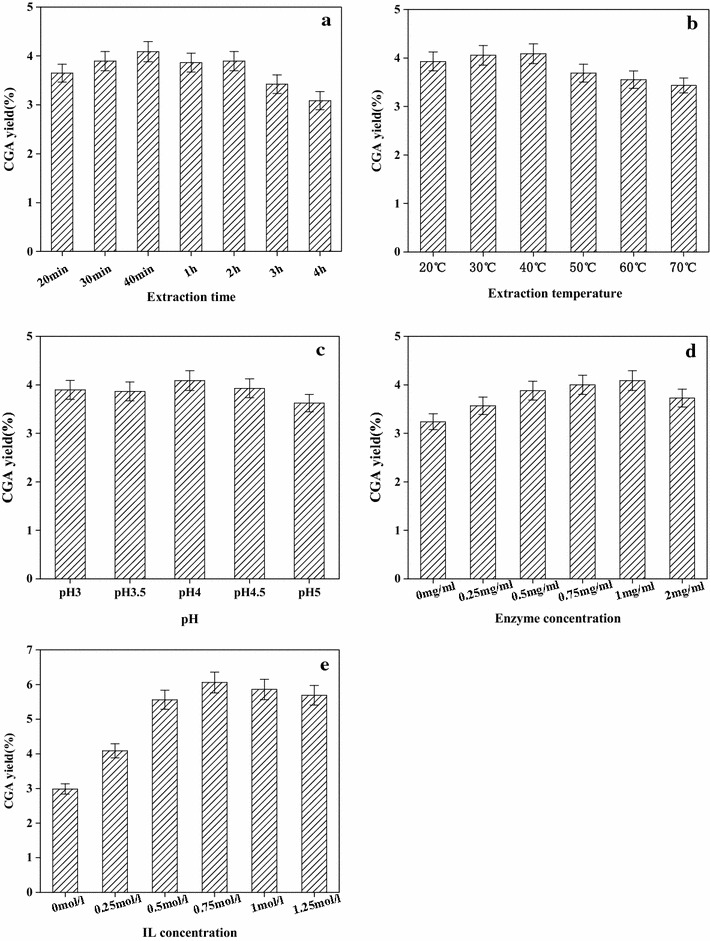
Effects of extraction time (**a**), extraction temperature (**b**), pH (**c**), enzyme concentration (**d**), IL concentration (**e**) on the extraction yields of CGA from *FLJ*

The extraction time considerably affected the extraction rate of CGA. Under the conditions of 0.5 mg/ml pectinase and 0.25 M [C4mim]Br treatment, pH 3.0, and temperature 40 °C, the influence of extraction time on yields was studied. The results in Fig. 3a illustrated that when the extraction time increased from 20 to 40 min, the extraction yields increased obviously. And yet the time was changed from 40 min to 4 h, the results remain essentially unchanged. This may be due to the isomerization of the ester groups in the GCA structure and also explained that the long extraction time would make GCA hydrolyzed, which would lead to the decreased GCA extraction yield. It was reported that pectinase can catalyze hydrolysis of ester linkage and using *Eucommia ulmoides* as the raw material and IL-pectinase as the extraction medium, the extraction rate of CGA was close to 0 under 4 h in 0.25 M [C4mim]Br aqueous solution. We have carried on the experiment to verify, and discovered that long periods of extraction time can cause CGA to be broken down by the HPLC result.

Therefore, 40 min was chosen as appropriate time for subsequent experiments.

### Effect of extraction temperature

To select a proper extraction temperature, we did a series of experiments to complete extraction process. The influence of extraction time on CGA extraction yield was studied under the conditions of 0.5 mg/ml pectinase and 0.25 M [C4mim]Br treatment, pH 3.0, and extraction time 40 min, As shown in Fig. 3b, the extraction yield reached the maximum at the optimum temperature of 40 °C and decreased with the increase of temperature. It may be the effect of temperature on the catalytic activity of the enzyme resulting in a decrease in the extraction rate of GCA.

### Effect of pH value

Holding the conditions of 0.5 mg/ml pectinase and 0.25 M [C4mim]Br treatment, extraction time 40 min and extraction temperature 40 °C, the influence of pH value on CGA extraction yield was investigated. As shown in Fig. 3c, pectinase showed ability to collapse within a wide pH range. It can be noticed that the extraction yields varied unregularly in the range of pH 3–5. This result showed the activity of pectinase was susceptible to solvent pH. The extraction yield of CGA reach peak value around pH 4.0.

### Effect of enzyme concentration

As shown in Fig. 3d, the influence of different enzyme concentrations on the extraction yields was investigated under the conditions of 0.25 M [C4mim]Br treatment, extraction time 40 min, pH 4.0, and temperature 40 °C. At the concentration of 1 ml/mg, the extraction yield reached the maximum, then the extraction yield of CGA was almost unchanged. Higher concentrations than 1 mg/ml couldn’t increase extraction yields. Thus, 1 mg/ml was chosen as appropriate concentration for the extraction process.

### Effect of IL concentration

As shown in Fig. 3e, under the conditions of 1 mg/ml pectinase treatment, pH 4.0, extraction time 40 min, and temperature 40 °C, the influence of different ionic liquid concentration on the yields of CGA was evaluated. Concentration of 0.75 mol/l gave a peak extraction yield about 6.06%, indicating that a concentration of 0.75 mol/l offered adequate amounts of pectinase for the disruption of cell wall. Higher concentrations than 0.75 mol/l did not increase yields. Thus, 0.75 mol/l was chosen as appropriate concentration for the extraction process.

### Comparison with the reference methods

In order to compare our method with the previous reported methods, some representative literatures data were listed in Table 1. Obviously, our method exhibits a good extraction efficiency and higher yields were obtained.

**Table 1:** Comparison of ILEAE with the reference and conventional methods

Method	Optimized extraction parameters	CGA yield (%)
WE	Liquid ratio 10 ml/g, extraction temperature 75 °C, extraction time 1 h	2.29 (Yu et al. 2010)
	Liquid ratio 15 ml/g, extraction temperature 70 °C, extraction time 1 h	3.8 (Li et al. 2011)
REE	Ethanol concentration 80% (v/v),extraction temperature 70 °C, extraction time 80 min	3.63 (Li and Hou 2013)
	Ethanol concentration 75% (v/v), flocculation time 24 min, reflux time 1.5 h	3.81 (Xiao and Li 2011)
UAE	Soaking time 12 h, ultrasonic time 45 min, ethanol concentration 60%	5.62 (Lu et al. 2015)
MAE	Microwave power 300 W, microwave time 3 min, extraction temperature 60 °C, ethanol concentration 70% (v/v), extraction time 20 min	5.16 (Cao et al. 2008)
EAE	Dosage of pectinase 0.5%, operating temperature 45 °C, extraction time 120 min	4.55
ILEAE	0.75 M [C6mim]Br, extraction time 40 min, extraction temperature 70 °C, pH 4.0, 1 mg/ml pectinase	6.06

### Scanning electron micrographs (SEM) of different samples

**Fig. 4:**
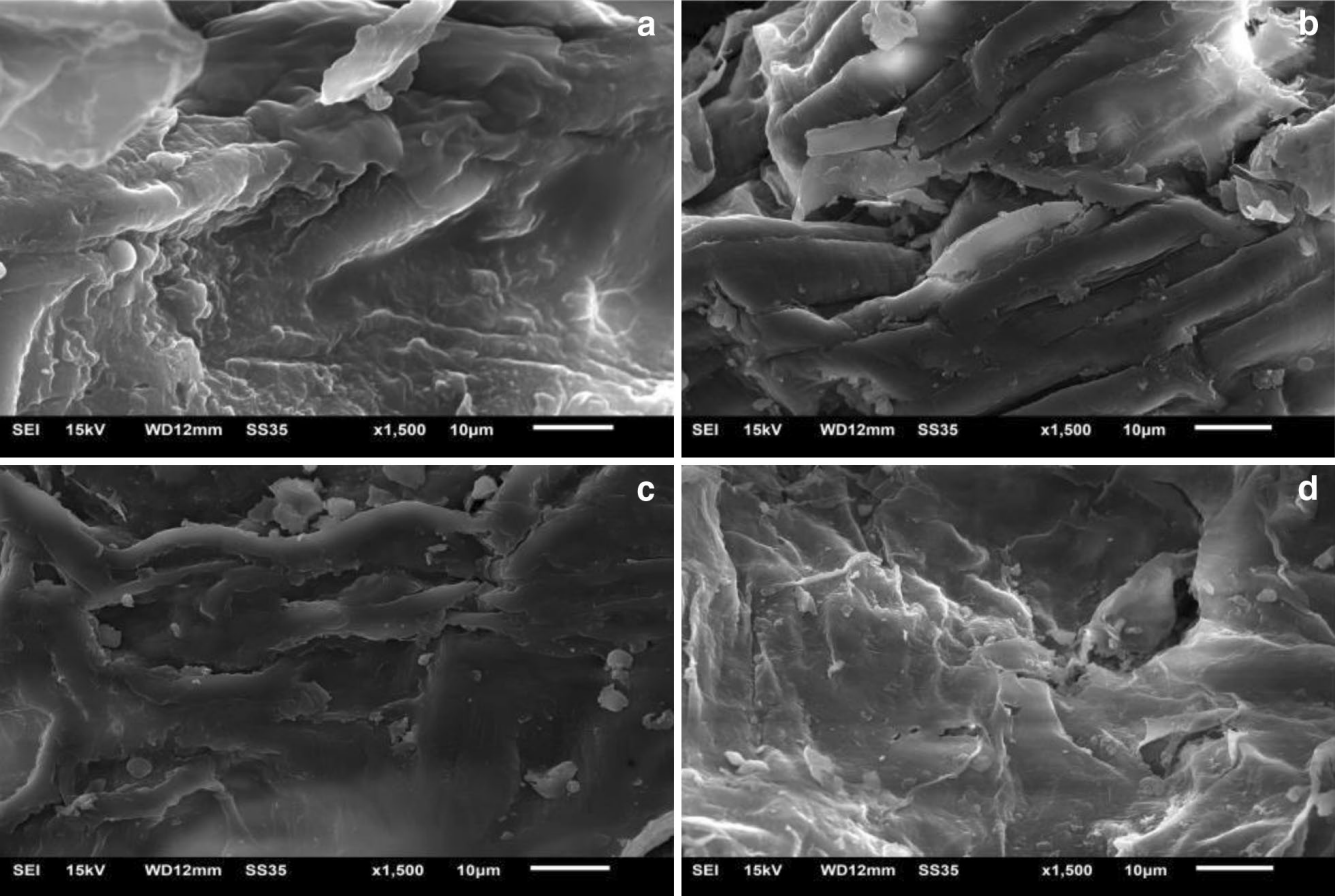
The scanning electron micrographs of *Flos Lonicera Japonicae*. Untreated sample (**a**), treated only with pectinase (**b**), treated only with ionic liquids (**c**) and treated with ILEAE (**d**)

In ILEAE method, the *FLJ* samples were detected by SEM for revealing the effect of ILEAE process and extraction mechanism on *FLJ* structure change. Figure 4 showed the micrographs of raw sample (a), the sample (b) treated only with pectinase, the sample (c) treated only with ionic liquids, and the sample (d) treated after ILEAE 2 h, respectively. From the sample (a), we found untreated samples showed a clear structure: thicker cell wall and intact cell structure. After pectinase treatment (b), the cell surface had undergone minor changes and some of the tissue damage occurred with protrusions in the middle of the leaves. From the sample (c), we found that after ionic liquid treatment, cell wall surface was significantly thinned, and the cell structure changed, which led to the target product exposed to the extraction solution. In the Fig. 4(d), after the mixed treatment of IL-enzyme, the cell wall surface tissue wrinkle serious and the cell wall thinner than a, b, c with the protrusions of the leaves disappeared. There is always a cell uplift in the leaves of the plant material, and the degree of protrusions in the treated sample can represent the dissolution and extraction capabilities of the liquid solution.

### Circular dichroism (CD) spectroscopy analysis

**Fig. 5:**
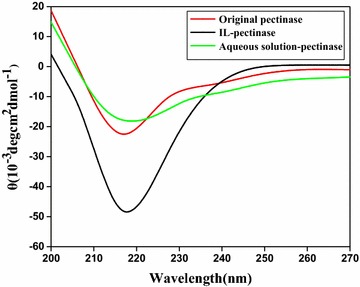
CD spectra of original pectinase, aqueous solution pectinase, and pectinase treated with ILs

Figure 5 showed the characteristic shape of the CD signal of the original pectinase, aqueous solution pectinase, and pectinase treated with ionic liquids which reflect the change of the secondary structure of the protein. Compared with the original pectinase, the sample treated with aqueous solution and the sample treated with ionic liquids had varying degrees of change on characteristic shape, which may cause changes of the secondary structure of the protein and the tertiary structure.

The results shown in Table 2 indicated the percentage change of α-helix, β-sheet, β-turn, and random crimp content in the secondary structure before and after pectinase treatment. Compared with the activity of the original pectinase, we found that the activity of aqueous solution pectinase decreased by 13.5% and the activity of IL- pectinase increased by 23.5%, respectively, and the CGA yield improved obviously. The results showed that the change of pectinase secondary structure resulted in enzyme activity increase which also make GCA extraction yield increased.

**Table 2:** The percentage of secondary structure elements and enzyme activity

Sample	α-helix (%)	β-sheet (%)	β-turn (%)	Random coil (%)	Enzyme activity (u/mg)
Original pectinase	11	33	20	36	100^a^
Aqueous solution pectinase	9.6	29.2	21	40.2	86.1^b^
IL-pectinase	20	19	24	37	123.5^c^

^a^Untreated

^b^Treated under a condition of extraction time 40 min, extraction temperature 70 °C, pH 4.0, 1 mg/ml pectinase, and the solution was aqueous solution

^c^Treated under a condition of 0.75 M [C6mim]Br, extraction time 40 min, extraction temperature 70 °C, pH 4.0, 1 mg/ml pectinase, and the solution was IL aqueous solution

## Conclusions

In this work of ILEAE, pectinase was successfully combined with ionic liquids for the extraction of CGA from *FLJ*. As far as we know, this is far-reaching significance investigation on developing a method for extraction of CGA from *FLJ* using ionic liquid-based enzyme-assisted solvent as extraction medium. From the final test results, the CGA yield was enhanced obviously with the addition of pectinase and ionic liquids. Compared to reported extraction methods, the optimal ILEAE method reduced the extraction time significantly and offered higher extraction yield. By taking into account the practical application, the ILEAE showed great prospect and provided new ideas for the extraction and separation of other natural products.
